# Supplementary material for: Effect of Sample Transportation on the Proteome of Human Circulating Blood Extracellular Vesicles
Source: Int J Mol Sci. 2022 Apr 19;23(9):4515. doi: 10.3390/ijms23094515 (PMC9099550; doi:10.3390/ijms23094515)
Supplement: Supplementary file 1 [file ijms-23-04515-s001.zip › ijms-1602167_supplMaterial_rev_edited.pdf]

Supplementary Materials

Effect of Sample Transportation on the Proteome of Human Circulating Blood Extracellular Vesicles

Uldry Anne-Christine[1,2], Maciel-Domiguez Anabel[1,2], Jornod Maïwenn[1,2], Buchs Natasha[1,2], Braga-Lagache Sophie[1,2], Justine Brodard[3], Jovana Jankovic[3], Bonadies Nicolas[2,3], Heller Manfred[1,2]*

[1] Proteomics and Mass Spectrometry Core Facility, Department for BioMedical Research (DBMR), University of Bern, Switzerland

[2] Department for BioMedical Research, University of Bern, Switzerland

[3] Department of Hematology and Central Hematology Laboratory, Inselspital, Bern University Hospital, University of Bern, Switzerland

*** Corresponding Author:**

Manfred Heller (PhD)

Department for BioMedical Research

Murtenstrasse 24

3008 Bern, Switzerland.

Phone: +41 31 684 04 82

Email: manfred.heller@dbmr.unibe.ch

Supplementary Materials

Supplementary Methods

Measurement of forces

The forces impacting on the samples during their transport from the venipuncture site to the wet lab were measured and recorded by a mini-computer *Raspberry Pi Zero,* equipped with a *SenseHat* (RPi0-SH) add-on board. The inertial measurement unit (IMU) on the RPi0-SH included an accelerometer, a gyroscope and a magnetometer. Calibration was performed at the receiving lab location. Calibration data is used by the *RTQF fusion algorithm* of the *RTIMULib* open-source library in order to report the orientation of the device with respect to a fixed Earth-bound reference frame. The orientation information is needed in order to determine the Earth gravitation vector in the device reference frame, and thereby reconstruct the accelerations within this reference frame. The IMU reports 3D accelerometer data and orientation in Euler angles at a sampling rate of 24 Hz. The size of the *RPi0-SH* is such that it fits exactly in the PTS tube; further packing arrangement largely prevented independent movements of S-Monovettes. For C transport the *RPi0-SH* was securely placed in the same rack as the S-Monovettes. One can therefore reasonably assume that the measured accelerations are representative of the forces acting on the S-Monovettes. The carrier placed the rack in a bag without further support, and the bag was held in one hand during the walk. It was intended that the carrier follows an identical route for all twelve transports; works along the set path forced however the carrier to take a small detour on two occasions. Both routes included an outdoor stretch, climbing one flight of stairs and the use of an elevator.

Calculation of transport metrics

Three transport metrics that encapsulate the history of shocks and vibrations impacting on a sample during a transport were calculated from the acceleration signal:

1. Teaker-Kaiser operator (TK), defined for discrete acceleration *a* at any time point *t*:

$$TK[a_{(t)}] = a_{(t)}^2 - a_{(t-1)} \times a_{(t+1)}$$

TK represents a measure for the energy of the acceleration signals. The values reported are the mean across the whole transport event.

2. Root Mean Square (RMS) acceleration over the event time interval Δ*T* is given by:

$$RMS = \sqrt{\left(\frac{1}{\Delta T}\right)\int_0^{\Delta T} a_{(t)}^2 \, dt}$$

RMS is interpreted as a measure of the total amount of acceleration undergone by the samples.

3. Vibration Dose Value (VDV):

$$VDV = \left(\int_0^{\Delta T} a_{(t)}^4 \, dt\right)^{\frac{1}{4}}$$

VDV is a measure of the vibration dose and is often used in the context of studying the impact of vibrations on biological tissues.

For each C transport a section of the signal showing regular oscillations over a period ranging from 42 to 172 s was isolated and Fourier transformed (fast Fourrier transformation applying a Hanning window).

cEV isolation protocol comparison

Blood from one of the donors of this study was drawn in the laboratory (no transportation involved) to prepare PFP aliquots of 400 µL as described in the main manuscript. cEV were either isolated with the centrifugation protocol (CEN) as described in the main manuscript or by size exclusion chromatography (SEC) as described in the following three protocols:

- *SEC_SN protocol*: PFP aliquots were centrifuged for 10 min at 2’000 g and 20°C as recommended by the SEC manufacturer (qEV, iZON Science, New Zealand). The supernatant (SN) was carefully removed by leaving approximately 50 µL in the tube and loaded on the SEC column.
- *SEC_P protocol*: PFP aliquots were centrifuged for 40 min at 16’000 g and 20°C. The supernatant was carefully removed leaving approximately 50 µL liquid in the tube to which 200 µL PBS were added by vigorous shaking. This EV suspension was loaded on the SEC column.
- *SEC_Pw protocol*: A cEV suspension was prepared as described above for the SEC_P protocol. This suspension was centrifuged again for 20 min at 16’000 g and 20°C. A volume of 200 µL from the supernatant was carefully removed and replaced by 200 µL PBS and vigorous shaking followed by a third centrifugation and resuspension of 50 µL pellet fraction in 200 µL PBS. The final EV suspension was loaded on the SEC column.

SEC columns were stored at 4°C, equilibrated to room temperature before use for one hour, and rinsed with 10 mL PBS at a flow rate of 1 mL/min. The cEV suspensions were loaded on the top of the column, let diffuse into the column bed and cEV were eluted by passing 10 mL PBS at a flow rate of 1 mL/min through the column. Fractions of 0.5 mL were collected in 1.5 mL polypropylene tubes, each containing 50 µL PBS and a PVDF membrane disk (0.2 µm, ThermoScientific) of 5 mm diameter cut into four to six small pieces. The PVDF membrane pieces were activated beforehand by incubation for 5 min in 200 µL methanol, followed by

0.05% SDS/5% methanol/0.05% DTT, and finally PBS. After collection of fractions, each tube was centrifuged for 1 hour at 3’000 g and 20°C. Proteins bound to PVDF pieces were on-membrane digested as described elsewhere [1], and eluted peptides analyzed by nLC-MS2 as described in the main manuscript. SEC columns were regenerated by passing subsequently 20 mL PBS, 10 mL 0.5 M sodium hydroxide, 30-50 mL PBS (bringing pH back to neutral), and 20 mL 20% ethanol for storage.

Nanoparticle tracking analysis (NTA) of SEC fractions

In order to estimate the cEV size distribution within the SEC fractions, fractions 5 to 9 produced with the same protocols as above, but without placing PVDF membranes in the collection tubes, were analyzed on a nanoparticle tracking instrument (Nanosight NS300, Malvern, Almelo, NL). Capture screen gain was set at 12-15 while the camera level was set at level 10-12. A standard measurement was performed with 5 captures of 60 sec each adjusting the flow rate such that 30-45 particles were captured per frame. Particle counts were averaged and graphically plotted in log2 scale with Matlab (version R2010a).

Supplementary Results

Non-consistent impact of transport method on individual cEV protein compositions: removal of outliers BE351 and BE140

It has already been noted that BE351 is an outlier of the ZetaView distributions in PPP as shown in Fig. 2 of the main manuscript. Furthermore, the differential expression analysis in PFP of C versus PTS for this donor revealed an exceptional number of proteins (304 against 0-1 for other donors) as significantly increased in PTS compared to C. There are fifteen cell-type specific proteins amongst those 304 BE351 PTS proteins, with thirteen being platelet-associated. That a potential problem of platelet activation occurred in the PFP PTS sample of BE351 was corroborated by the consistently high log2fc of platelet-specific cell markers CD41 and CD62P (red bars in sFig. 3). The two lymphocyte/monocyte-specific proteins CD40 and CD102 were also higher in PTS, while

the opposite was found for erythrocyte-, macrophage and endothelial-specific proteins (CD233, CD14, and HSPG2). Intriguingly, hardly any change in intensity between PTS and C was detected with the BE351 PPP isolates prepared from the very same whole blood input material like PFP samples (blue bars in sFig. 3). A GO term enrichment analysis (not shown) revealed that collagen-containing extracellular matrix was enriched in the non-changed protein fraction, while terms like focal adhesion or cytoskeleton were enriched in PTS isolates of PFP BE351. We interpret these observations as damage to cEV during the preparation of PFP aliquots from the carrier transported blood of donor BE351.

As for BE140, there were 1132 proteins in the PFP C isolate, which exceeded by far the numbers quantified in PFP PTS or both PPP samples, with 869, 907 and 900, respectively. A GO term over-representation analysis (not shown) submitting the 271 on-off proteins seen in the PFP C isolate revealed that proteins lost in PTS were of cellular origin. Again, we have to assume that something happened during the preparation of the different plasma aliquots, confounding the measurement outcome.

Comparison of cEV isolation methods

Our centrifugation based cEV isolation protocol (CEN) outperformed any of the tested SEC protocols in terms of measured intensities (sFig. 4), repeatability (R.S.D. in sFig. 4), and quantified numbers (sFig. 5) of proteins with a cellular origin, including cell type specific proteins, considered to represent the recovery of cEV from blood plasma. The proteome analysis of cEV isolated with protocol SEC_SN, as recommended by the manufacturer of the SEC columns, revealed that cellular proteins started to elute in fraction 5, reaching a maximum in intensity in fraction 6 and 7, then dropping back to an intensity level as measured in fraction 5 in fraction 8 and below in 9 (sFig. 4). Blood plasma proteins, including apolipoproteins, coagulation and complement factors as well as immunoglobulins eluted already in fraction 5 with slightly increasing intensities up to fraction 9, while other serum proteins, for instance albumin, eluted at maximum intensity in fraction 5, decreasing until fraction 8, and increasing again in fraction 9 (sFig. 4). When PFP was first centrifuged to sediment cEV, and the pre-concentrated/purified cEV were fractionated by SEC (protocol SEC_P), the overall intensities of cellular proteins increased in fractions 6 to 9 compared with SEC_SN together with a changed

elution profile showing a maximum intensity in fraction 7, then dropping back to a level as in fraction 5 only in fraction 9. The elution profile of all contaminating blood plasma proteins showed a steady increase from fraction 5 to 9. Repeating the cEV sedimentation with two more centrifugation steps, as done in the CEN protocol followed by SEC (SEC_Pw), the blood plasma protein elution profile did not change compared to SEC_P, albeit the overall intensities for apolipoprotein, coagulation and complement factors, and immunoglobulins were decreased compared with the other two SEC protocols, while other serum proteins were still present at similar levels with the exception of fraction 5. The cellular protein elution profile resembled the one of protocol SEC_P, albeit reaching higher intensities in fractions 7 to 9. The sedimentation of cEV by centrifugation prior to SEC in protocols SEC_P and SEC_Pw was meant to remove blood plasma proteins, which was indeed achieved. The additional washing steps with protocol SEC_Pw could however not increase the number of quantified cellular proteins, but decreased slightly the number of blood plasma contaminations, which might explain increased cellular protein intensities due to a decreased suppression by plasma proteins (sFig. 5). This suppression effect does likely explain the decreased cellular protein recovery seen with the SEC_SN protocol (sFig. 4 and 5). The abundant cell type specific proteins CD233 (representing cEV of erythrocyte origin) and CD61 (platelet origin) had a very similar elution profile with all three SEC protocols, reaching a maximum in fractions 6 and 7 (sFig. 4). The recovery of the lower abundant markers CD14 (macrophage origin) and HSPG2 (endothelial cell origin) was clearly compromised by the SEC protocols in an inconsistent manner, as a complete loss of CD14 or HSPG2 in SEC_P or SEC_Pw protocols, respectively, occurred, while both could be detected with SEC_SN. Considering all elution profiles, we concluded that SEC fractions 6 to 8 contained the purest cEV, for which reason we used the proteins quantified in these fractions to compare the cEV proteome with the CEN one (Venn diagrams in sFig. 5). We could quantify a total of 127 cellular proteins in the combined set of 9 SEC fractionations, which were not found in the three replicates using our CEN protocol. However, the latter produced 151 proteins, which were nowhere found with any SEC preparation. With the contaminating blood plasma protein this relation was converted to the opposite, with 27 proteins not found and only eight found exclusively with the CEN protocol.

Our results demonstrate clearly that SEC does not provide the means for an improved purification of cEV from blood plasma proteins or lipoproteins compared with our sequential centrifugation protocol. In fact, when using the SEC columns as

recommended by the manufacturer, a significant reduction of cEV purification specificity is encountered compared to our isolation protocol, as the ratio of cellular proteins to blood plasma proteins is decreased (relative protein number column graph of sFig. 5). In addition, immunoglobulins represent an important source of contamination in the SEC fractions of protocols SEC_SN and SEC_P. NTA of the cEV-enriched SEC fractions collected with protocol SEC_SN revealed a similar mean particle size between 90-123 nm and a similar size distribution between the first and 99th percentile of particle counts in all four fractions five to eight (sFig. 6). The peak maxima were shifted by about 10 nm to higher values with the SEC_P protocol and the size distribution increased especially in fraction 5 and 6, which would indicate an isolation of larger EV in those early fractions, as is the expected outcome from SEC. Washing cEV further with additional centrifugations (protocol SEC_Pw), resulted in increasing particle size peak maxima from fraction 5 to 8, shifting from 90 to 160 nm, and again a larger size distribution in fractions 5 to 7 compared with SEC_SN. This result is unexpected, as the particle size in later SEC fractions should become smaller, not bigger. We can only speculate that the removal of contaminating blood plasma proteins by prior washing steps in protocol SEV_Pw does induce a vesicle clotting effect, which is more pronounced with smaller cEV eluting in later fractions where the overall number of cEV is bigger based on the intensities of cellular proteins as illustrated in sFig. 4. This is corroborated when comparing the effect on the size distribution between protocols SEC_SN and SEC_P, and with the results on NTA measurements made directly in PFP and PPP as shown in the main manuscript.

Supplementary Tables

Table S1. Clinical characteristics of study participants.

Donor	Diagnosis*	Age / gender	Ec (10^{12}/L)	Tc (10^9/L)	Lc (10^9/L)	Gc (10^9/L)	Mc (10^9/L)	Ly (10^9/L)	HGB (g/L)	CRP (mg/L)	Tranfusion dependency
BE349	Healthy	47 / m	5.63	230	5.29	2.93	0.51	1.85	169	-	-
BE351	Healthy	56 / m	5.07	165	3.74	2.63	0.32	0.79	151	-	-
BE352	Healthy	46 / f	5.20	278	6.94	4.77	0.41	1.76	144	-	-
BE353	Healthy	39 / m	5.51	177	7.24	4.22	0.68	2.34	161	-	-
BE354	Healthy	42 / f	4.66	269	11.15	6.30	1.11	3.74	140	-	-
BE355	Inactive spondyloarthritis	45 / f	4.00	200	11.51	7.44	1.10	2.97	128	-	-
BE019	MDS, hypoplastic	31 / m	2.48	105	2.03	2.24	0.02	0.77	83	5	-
BE009	MDS, lower risk	79 / m	3.53	168	1.72	1.93	0.40	0.95	124	<3	-
BE322	MDS, lower risk	32 / m	3.39	276	4.7	3.86	0.07	0.77	125	28	-
BE025	Aggressive systemic mastocytosis with MDS, lower risk	81 / m	2.31	125	1.31	1.73	0.09	0.29	66	18	high
BE140	MDS, higher risk	82 / f	3.49	611	7.12	8.24	0.34	1.12	88	-	low
BE363	Secondary AML	53 / m	3.08	149	5.2	4.58	0.53	0.89	80	61	low

*MDS: Myelodysplastic Syndromes; AML: Acute Myeloid Leukaemia.

Table S2. Qualitative summary of the g-forces experienced by S-Monovettes during transportation.

**Donor**	**Transport duration [min]**		**Max [g]**		**Mean [g]**		**S.D. [g]**		**Median [g]**		**Skewness**		**# of shocks ≥ 2.5g**	
	**C**	**PTS**	**C**	**PTS**	**C**	**PTS**	**C**	**PTS**	**C**	**PTS**	**C**	**PTS**	**C**	**PTS**
BE349	5.57	2.15	0.7	21.1	0.2	0.9	0.1	1.3	0.1	0.4	1.0	4.4	0	115
BE351	5.29	2.20	0.8	23.6	0.2	0.9	0.1	1.4	0.1	0.4	0.9	5.3	0	116
BE352	5.18	2.66	0.8	22.5	0.2	0.7	0.2	1.3	0.2	0.2	0.8	5.4	0	105
BE353	5.14	2.55	0.8	23.7	0.2	0.7	0.2	1.2	0.2	0.3	0.8	5.1	0	118
BE354	6.47	2.69	0.7	22.3	0.2	0.7	0.1	1.2	0.1	0.3	0.9	5.1	0	113
BE355	5.54	3.10	0.9	23.0	0.2	0.6	0.1	1.2	0.2	0.2	1.0	5.3	0	114
BE009	5.22	2.17	0.9	23.9	0.2	1.0	0.2	1.5	0.2	0.5	0.7	5.8	0	111
BE019	5.21	2.11	0.8	22.7	0.2	0.9	0.1	1.3	0.2	0.3	0.7	4.6	0	112
BE025	5.37	2.32	0.9	19.7	0.2	0.8	0.2	1.3	0.2	0.3	0.9	4.4	0	117
BE140	5.82	2.15	2.3	17.2	0.2	1.0	0.1	1.4	0.1	0.5	2.3	4.0	0	123
BE322	4.69	2.20	1.3	22.3	0.2	0.9	0.2	1.4	0.2	0.4	0.7	4.4	0	130
BE363	6.44	2.29	0.8	22.6	0.2	0.9	0.2	1.2	0.1	0.4	0.9	4.6	0	115
Mean	***5.50***	***2.38***	***1.0***	***22.1***	***0.2***	***0.8***	***0.2***	***1.3***	***0.2***	***0.4***	***1.0***	***4.9***	***0.00***	***116***
R.S.D.	9.6%	12.8%	45.7 %	8.7 %	0.0%	15.6 %	34.8 %	7.6 %	32.5 %	28.6 %	44.8 %	11.1 %		5.4%

Table S3. Calculated transport metrics.

Donor	Mean of Teaker-Kaiser operator [g²]		Root Mean Square (RMS) [g]		Vibration Dose Value (VDV) [$ms^{-7/4}$]	
	C	PTS	C	PTS	C	PTS
BE349	0.01	0.87	0.21	1.58	1.15	12.09
BE351	0.01	1.12	0.20	1.66	1.07	13.78
BE352	0.02	0.83	0.29	1.48	1.52	13.42
BE353	0.02	0.72	0.27	1.43	1.42	12.50
BE354	0.01	0.72	0.20	1.40	1.15	12.37
BE355	0.01	0.68	0.21	1.37	1.16	12.97
BE009	0.03	1.32	0.27	1.82	1.47	15.36
BE019	0.02	0.95	0.25	1.57	1.32	12.34
BE025	0.03	0.82	0.28	1.48	1.54	11.66
BE140	0.01	0.92	0.19	1.69	1.25	12.09
BE322	0.03	1.02	0.30	1.70	1.55	12.97
BE363	0.02	0.85	0.24	1.51	1.39	12.09
Mean	***0.02***	***0.90***	***0.24***	***1.56***	***1.33***	***12.80***
R.S.D.	45.5%	20.3%	16.2%	8.8%	13.0%	7.9%

Table S4. Number of quantified cEV-associated protein groups per donor and plasma type, with the corresponding numbers of (persistently) significantly over-expressed protein groups between transport types or uniquely found in either of them.

Donor	PFP					PPP				
	quantified	significant		uniquely in		quantified	significant		uniquely in	
	total	C	PTS	C	PTS	total	C	PTS	C	PTS
BE349	955	0	0	11	58	1755	0	0	53	30
BE351	1184	1	304	7	584	1456	0	0	41	8
BE352	1022	0	0	21	30	1651	0	0	113	10
BE353	969	0	0	63	31	1472	0	0	13	75
BE354	569	1	5	6	105	1615	0	0	7	129
BE355	903	0	0	12	42	1614	0	0	11	281
BE009	1342	0	0	26	28	1765	0	0	16	72
BE019	852	0	0	14	54	1722	0	0	12	36
BE025	918	0	0	164	9	1598	0	0	18	84
BE140	1140	0	0	271	8	931	0	0	31	24
BE322	1360	0	0	21	157	1545	0	0	24	16
BE363	841	1	0	148	8	1404	0	0	22	98
Median	***962***	***0***	***0***	***21***	***36.5***	***1606***	***0***	***0***	***20***	***54***
Min	***569***	***0***	***0***	***6***	***8***	***931***	***0***	***0***	***7***	***8***
Max	***1360***	***1***	***304***	***271***	***584***	***1765***	***0***	***0***	***113***	***281***

Table S5. Matrix of quantified features used for Spearman rank correlations used for Fig. 4 in main text.

	PFP	C												PTS											
	Quantified feature	BE349	BE351	BE352	BE353	BE354	BE355	BE009	BE019	BE025	BE140	BE322	BE363	BE349	BE351	BE352	BE353	BE354	BE355	BE009	BE019	BE025	BE140	BE322	BE363
ZetaView®	Particle conc.	5.5E+07	1.1E+08	4.0E+07	6.3E+07	1.1E+08	7.1E+07	6.5E+07	4.6E+07	2.9E+07	7.1E+07	4.7E+07	8.2E+07	7.4E+07	1.4E+08	8.2E+07	1.1E+08	1.3E+08	7.2E+07	8.8E+07	8.5E+07	5.9E+07	7.4E+07	1.0E+08	8.2E+07
	Original particle conc.	2.0E+12	2.3E+12	6.3E+11	1.0E+12	2.2E+12	1.1E+12	1.1E+12	7.6E+11	4.4E+11	1.4E+12	8.1E+11	1.3E+12	1.3E+12	2.4E+12	1.5E+12	2.0E+12	2.2E+12	1.3E+12	1.6E+12	1.5E+12	1.1E+12	1.3E+12	1.8E+12	1.5E+12
	Particle vol.	3.7E+19	3.2E+19	8.9E+18	1.5E+19	3.1E+19	1.4E+19	2.0E+19	1.3E+19	1.5E+19	2.0E+19	1.9E+19	2.4E+19	5.2E+19	7.5E+19	1.0E+20	1.3E+20	9.6E+19	6.3E+19	1.0E+20	1.3E+20	8.6E+19	6.6E+19	7.2E+19	8.4E+19
Blood cell count	Ec (10^12/L)	5.63	5.07	5.2	5.51	4.66	4	3.53	2.48	2.31	3.49	3.39	3.08	5.63	5.07	5.2	5.51	4.66	4	3.53	2.48	2.31	3.49	3.39	3.08
	Tc (10^9/L)	230	165	278	177	269	200	168	105	125	611	276	149	230	165	278	177	269	200	168	105	125	611	276	149
	Lc (10^9/L)	5.29	3.74	6.94	7.24	11.15	11.51	1.72	2.03	1.31	7.12	4.7	5.2	5.29	3.74	6.94	7.24	11.15	11.51	1.72	2.03	1.31	7.12	4.7	5.2
	Gc (10^9/L)	2.93	2.63	4.77	4.22	6.3	7.44	1.93	2.24	1.73	8.24	3.86	4.58	2.93	2.63	4.77	4.22	6.3	7.44	1.93	2.24	1.73	8.24	3.86	4.58
	Mc (10^9/L)	0.51	0.32	0.41	0.68	1.11	1.1	0.4	0.02	0.09	0.34	0.07	0.53	0.51	0.32	0.41	0.68	1.11	1.1	0.4	0.02	0.09	0.34	0.07	0.53
	Ly (10^9/L)	1.85	0.79	1.76	2.34	3.74	2.97	0.95	0.77	0.29	1.12	0.77	0.89	1.85	0.79	1.76	2.34	3.74	2.97	0.95	0.77	0.29	1.12	0.77	0.89
	HGB (g/L)	169	151	144	161	140	128	124	83	66	88	125	80	169	151	144	161	140	128	124	83	66	88	125	80
Force	TK	0.01	0.01	0.02	0.02	0.01	0.01	0.03	0.02	0.03	0.01	0.03	0.02	0.87	1.12	0.83	0.72	0.72	0.68	1.32	0.95	0.82	0.92	1.02	0.85
	RMS	0.21	0.20	0.29	0.27	0.20	0.21	0.27	0.25	0.28	0.19	0.30	0.24	1.58	1.66	1.48	1.43	1.40	1.37	1.82	1.57	1.48	1.69	1.70	1.51
	VDV	1.15	1.07	1.52	1.42	1.15	1.16	1.47	1.32	1.54	1.25	1.55	1.39	12.09	13.78	13.42	12.50	12.37	12.97	15.36	12.34	11.66	12.09	12.97	12.09
Summed log2-transformed iTop3 intensities for protein class or cell marker	cellmembrane	6629.1	3841.0	7916.0	7129.4	2870.2	6308.6	10467.0	5742.6	6772.4	8281.0	8753.5	6605.7	7081.1	9353.0	8060.6	6825.0	3793.9	6586.1	10567.8	6339.6	5136.1	5990.5	10221.0	4970.2
	cellpart	8946.9	4693.7	9820.6	9159.0	3251.8	7959.1	14205.7	7562.3	9701.4	12272.6	13686.2	7827.6	9758.4	12543.3	10099.9	8553.3	4582.1	8597.3	14202.9	8168.7	7578.8	8587.9	15499.3	5884.0
	cellsurface	1677.0	1529.2	1714.1	1728.7	1352.9	1756.9	2083.0	1658.4	1743.7	1816.0	1847.9	1726.2	1787.4	1932.8	1761.6	1752.0	1511.6	1785.2	2105.4	1707.4	1618.3	1653.0	1951.8	1643.6
	keratin	69.7	144.1	51.2	43.7	134.7	121.9	177.3	178.7	115.3	138.5	160.8	182.1	97.3	182.2	175.7	164.7	119.5	91.5	156.3	159.4	119.1	137.4	176.8	140.7
	coagulation factors	573.9	593.9	569.4	570.3	547.1	534.9	579.0	529.8	538.1	521.3	588.7	574.3	572.8	587.9	568.1	567.6	566.0	527.2	600.3	529.4	523.7	527.1	574.9	574.1
	apolipoprotein	525.9	549.3	514.7	527.2	583.0	508.3	507.4	510.1	456.5	546.3	533.1	486.2	554.1	529.7	516.3	530.8	570.8	507.8	509.5	511.3	482.1	571.6	509.5	489.4
	complement factors	575.6	608.0	571.0	551.0	570.1	604.8	579.7	572.8	577.7	551.4	561.9	653.9	578.8	578.3	545.8	555.0	562.0	597.8	578.9	566.9	584.5	560.5	535.1	666.4
	immunoglobulins	993.9	1216.6	1051.0	1520.0	988.0	1691.8	1147.2	1241.5	936.9	1332.8	928.0	1158.5	965.0	1163.3	1039.7	1598.7	963.5	1602.4	1074.4	1277.5	903.3	1254.8	901.4	1121.0
	other plasma proteins	1198.7	1241.3	1190.0	1167.6	1239.6	1158.6	1167.8	1197.8	1153.9	1083.7	1257.7	1236.9	1230.5	1258.2	1211.9	1204.7	1247.2	1167.4	1208.7	1204.9	1195.5	1098.5	1252.1	1256.6
	Exosome	12154.2	9103.5	12630.7	12740.2	7837.1	11447.3	15798.7	10978.4	12307.5	14043.5	15248.1	11812.1	12870.8	14862.8	12979.3	12260.9	8887.9	11902.0	15726.8	11504.9	10636.5	11599.1	16248.3	10439.4
	Blood microparticle	2814.8	2833.7	2701.1	2909.8	2754.9	2900.5	2770.8	2826.1	2725.6	2792.2	2803.1	2814.1	2783.5	2805.3	2710.2	2960.1	2758.7	2883.1	2811.5	2856.7	2703.7	2780.2	2751.7	2833.5
	Endothial cell	614.8	534.9	647.3	673.0	417.3	583.0	733.7	523.6	546.1	559.9	655.5	650.7	626.4	750.6	692.3	663.4	484.8	586.2	741.2	520.3	440.1	484.8	692.3	563.3
	Stem/Progenitor	524.7	472.9	556.8	605.7	356.8	486.6	617.0	479.1	409.6	461.8	561.3	596.8	540.7	639.5	604.6	599.9	396.1	516.8	627.5	474.8	331.4	392.1	594.0	495.1
	T Cell	588.8	544.0	654.4	703.7	461.5	618.1	675.0	527.8	487.8	572.2	620.5	725.9	600.5	721.5	679.7	722.5	474.6	616.9	684.0	550.8	406.3	512.2	652.5	642.7
	B Cell	517.7	472.6	584.6	607.3	412.1	546.4	602.6	461.0	420.3	522.9	550.9	607.0	527.1	644.8	609.6	628.7	446.5	545.4	612.1	455.8	381.0	467.2	582.2	528.0
	NK cell	444.9	366.0	470.3	534.7	346.5	452.3	504.7	367.4	338.5	403.8	452.2	559.1	454.2	507.6	491.9	534.3	356.6	451.8	510.7	380.5	284.5	392.2	485.3	502.0
	Lymphocytes	632.7	564.6	699.4	746.7	461.5	640.9	723.4	573.5	538.0	619.6	668.7	769.4	644.7	768.1	726.5	765.8	497.1	640.7	733.5	575.9	451.7	536.8	702.0	666.9
	Dendritic cell	230.0	181.2	272.7	278.0	166.3	235.6	284.3	204.4	151.0	228.9	238.6	325.6	237.4	289.7	295.3	255.9	167.7	234.8	286.4	187.2	142.1	159.9	264.1	281.6

	PFP	C												PTS											
	**Quantified feature**	**BE349**	**BE351**	**BE352**	**BE353**	**BE354**	**BE355**	**BE009**	**BE019**	**BE025**	**BE140**	**BE322**	**BE363**	**BE349**	**BE351**	**BE352**	**BE353**	**BE354**	**BE355**	**BE009**	**BE019**	**BE025**	**BE140**	**BE322**	**BE363**
	Monoc./Macroph.	695.9	628.2	784.0	832.1	543.5	706.5	807.7	640.5	602.5	676.6	754.1	872.4	708.5	852.6	813.0	851.9	603.6	704.9	819.1	635.9	538.1	619.5	788.2	788.5
	Granulocyte	433.9	392.7	494.5	586.8	354.3	500.9	487.9	441.4	372.1	437.5	462.7	615.6	440.8	511.1	502.2	591.1	383.2	504.3	493.6	458.4	338.0	406.3	491.5	625.4
	Leukocyte	739.4	669.4	828.8	874.9	543.5	770.6	853.6	680.5	649.0	722.6	798.2	916.9	753.3	900.4	857.4	895.9	603.6	770.2	865.8	680.8	560.8	639.6	833.7	832.2
	Platelet	715.2	608.8	757.6	749.5	455.9	707.8	815.8	657.3	658.1	700.8	755.3	729.7	732.7	795.5	766.1	720.8	573.5	714.0	824.5	686.5	606.8	616.4	779.9	613.3
	Erythrocyte	285.3	288.8	307.3	318.6	211.7	240.6	338.5	237.0	200.2	239.4	291.9	293.1	293.2	339.9	311.3	319.8	220.7	269.3	344.8	249.0	171.3	235.8	294.1	259.5

Table S6. Remaining non-zero protein coefficients with pure *lasso* (**C** $_{\alpha=1}$), or the *elastic net* (**C** $_{\alpha=0.5}$) approach. Proteins forming an interaction node in a string network as shown in Fig. 5-7 of the main manuscript are marked with a plus (+) sign.

**Gene Name**	**SwissProt Accession**	**C** $_{\alpha=1}$	**C** $_{\alpha=0.5}$	**Network C_TK/PTS/VDV**	**Network PTS_TK/RMS**	**Network PTS_VDV**
OIT3	Q8WWZ8	-1.8				
C4BPB	P20851-2	-1.5				
CORO1A	P31146	-1.3		+		+
ATP5PF (ATP5J)	P18859-2	-0.5		+	+	+
ST6GAL1	P15907	-0.3	-0.004			
HSPA1A	P0DMV8		-0.2	+	+	+
EFEMP1	Q12805-2	-0.05				
CFHR1	Q03591	0.06				+
KRT1	P04264	0.6				+
FLOT1	O75955	2.0		+	+	+
HRG	P04196	2.0		+	+	+
SERPINC1	P01008	3.6	0.3	+	+	+

Supplementary Figures

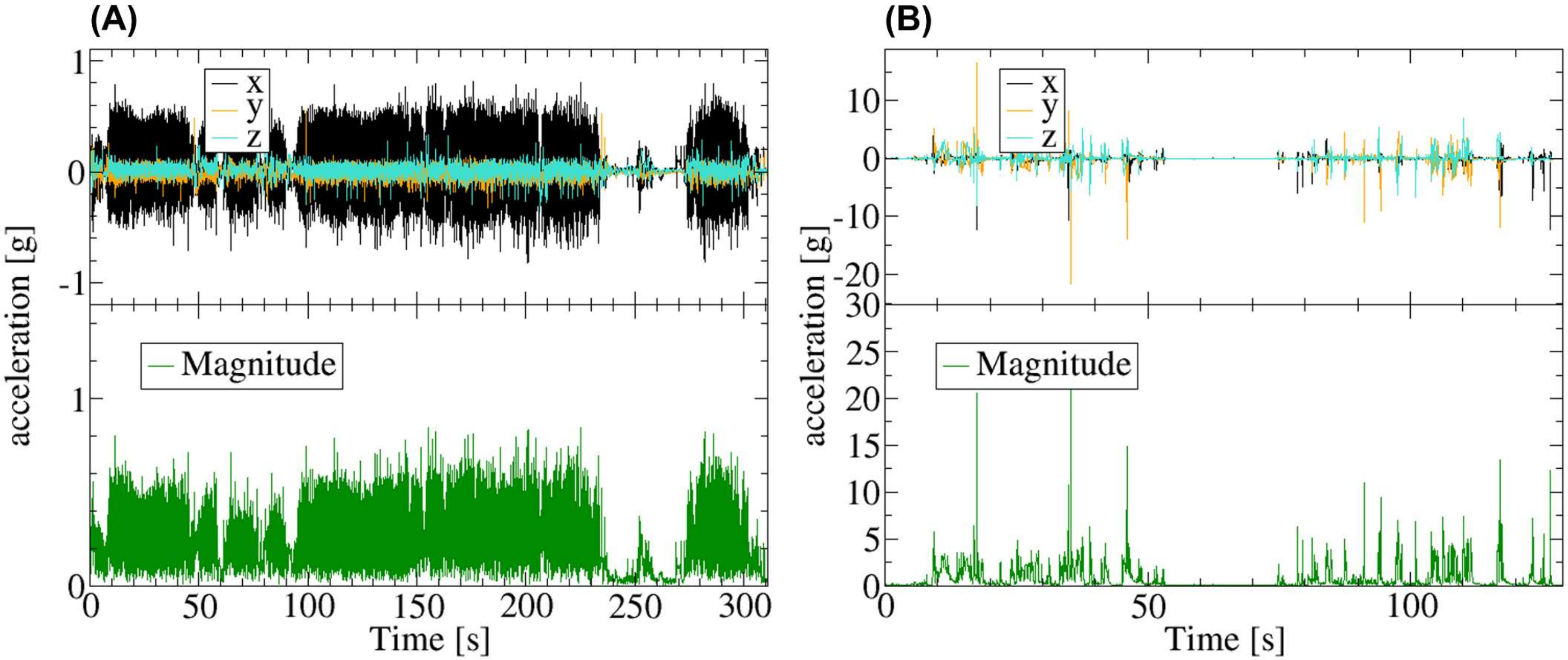

Figure S1. Accelerations measurement for one representative sample (BE352). The acceleration in the device reference frame is shown on the vertical axis in units of *g*, with the three components in the upper panels, and the magnitude in the lower panels. Panel (A) shows the C transport, panel (B) the PTS transport of this donor' samples. Note the substantially higher acceleration values exhibited in PTS. The C transport lasted for this sample 5.2 min, while the PTS transport was shorter with 2.7 min.

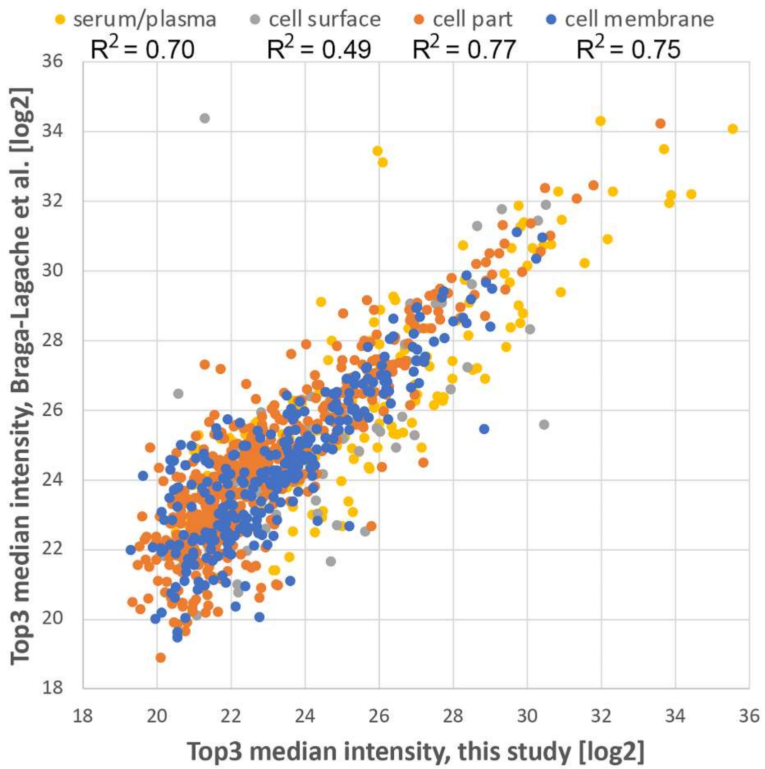

Figure S2. Protein intensity correlation per protein class. The median Top3 cEV protein intensities of proteins quantified in a former study by Braga-Lagache et al with the 400-µL aliquots of twelve healthy donors [2] on the y-axis and this PFP dataset on the x-axis were compared. Only proteins that were quantified at least three times in each dataset (N = 1009) were used. Each dot represents one protein and proteins were grouped by protein calls as indicated at the top of the scatterplot. Below each protein class the squared linear regression correlation is indicated. The raw mass spec data of Braga-Lagache et al was reprocessed through the exact same pipeline as the data in this study and is available on the pride ProteomeXchange server with identifier PXD003935. Please note that the data was acquired on two different mass spectrometers in the two studies, which might explain the shift to higher intensities in Braga-Lagache et al.

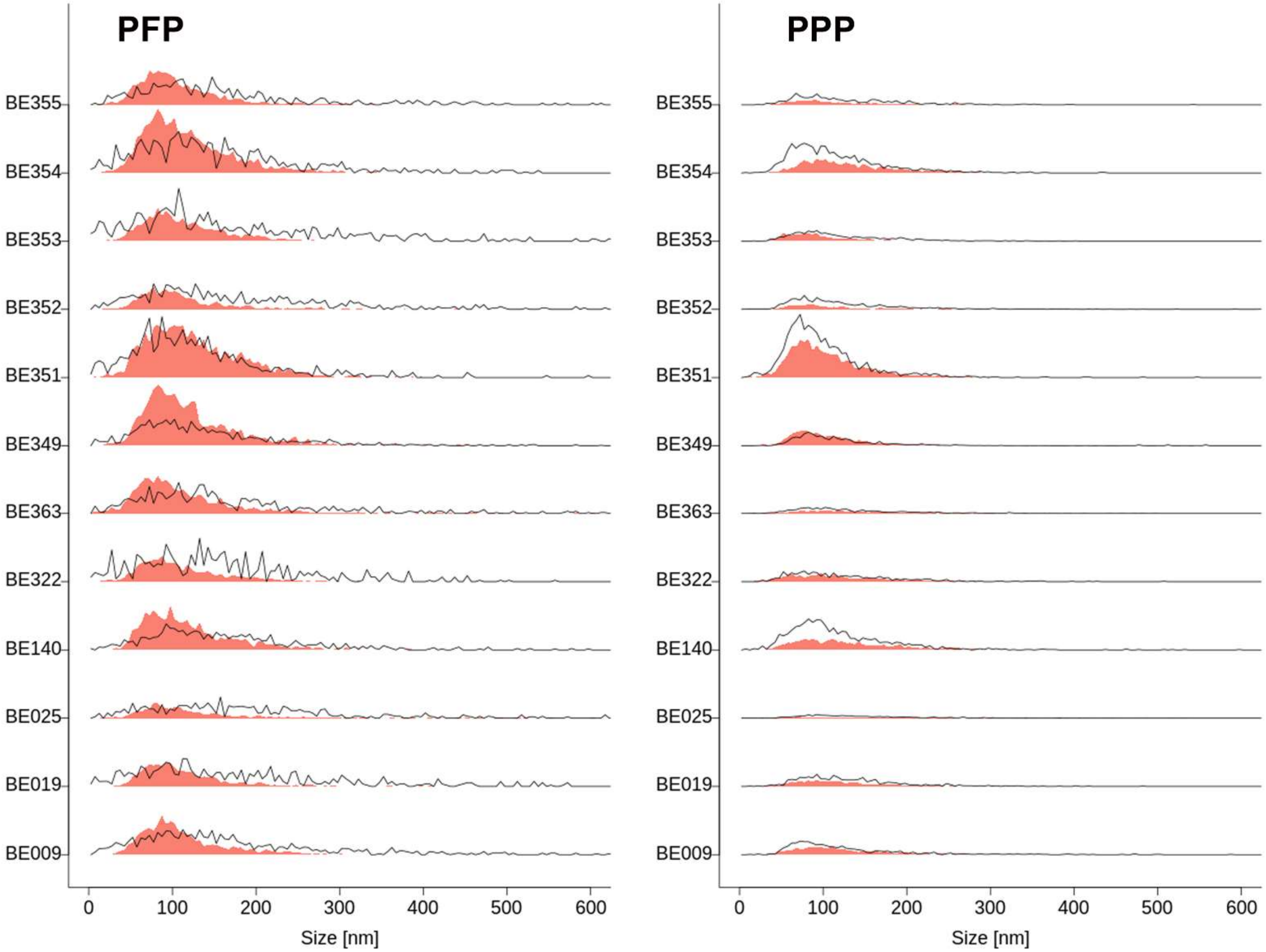

Figure S3. Nanoparticle size distribution in individual plasma samples. (A) Plot of the binned, averaged (over technical replicates) nanoparticle size distributions for PFP (left) and PPP (right). Particle size is in nm on the x-axis and counts on the y-axis (same scale everywhere). The red-filled curves illustrate data acquired from C samples, the black contures the ones from PTS, respectively.

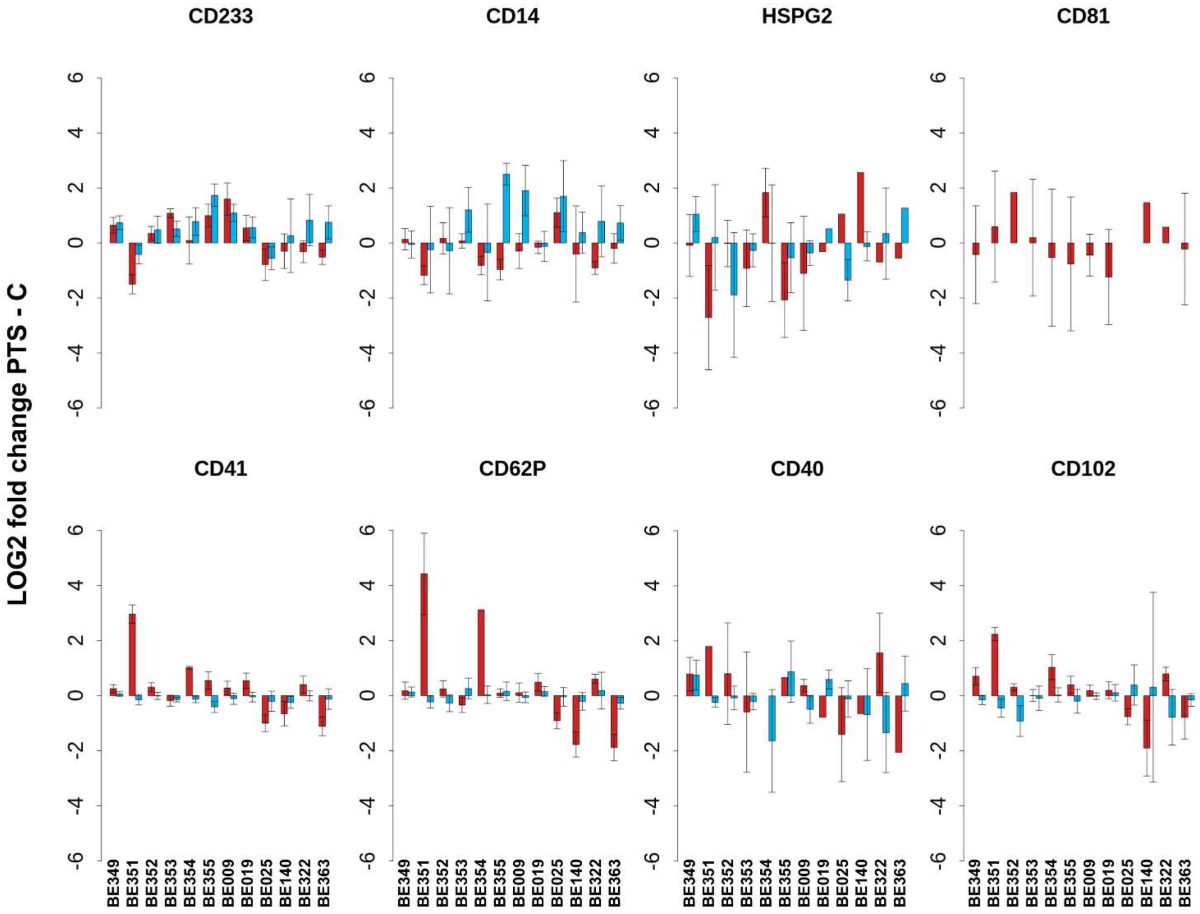

Figure S4. Relative change of cell-type specific protein intensities. The mean log2-fold change (y-axis, same scale on every plot) between PTS and C for cell-type specific protein intensities are shown as bar plots. The same marker proteins were used as in Fig. 3 of the main manuscript. Donors are indicated on the x-axis. The red bars represent the mean changes of the cEV isolates from PFP, the blue ones from PPP, and the whiskers extend one pooled standard deviation from the three replicates measured for each transport mode and donor. Protein intensities have been normalized separately in PFP and PPP.

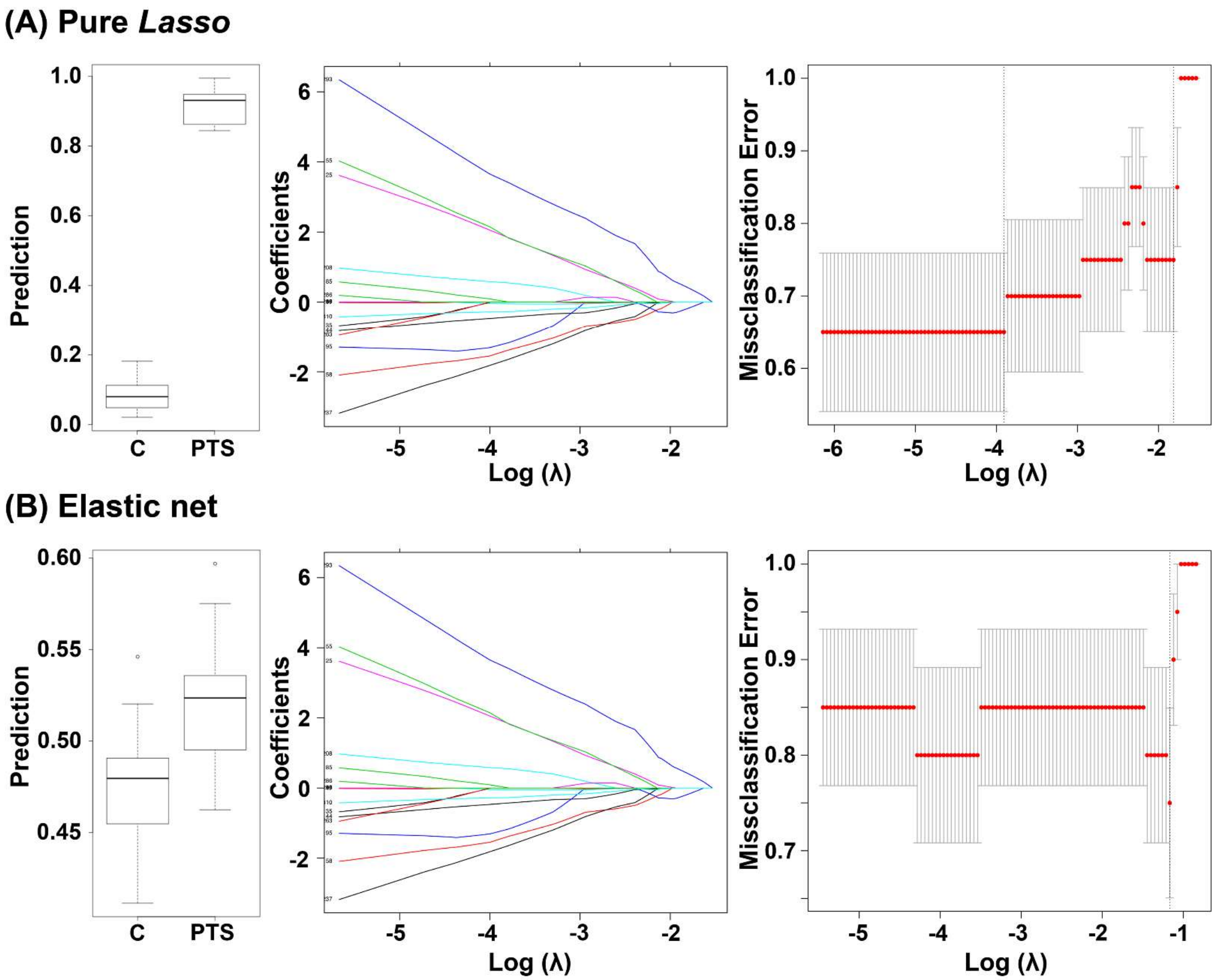

Figure S5. Lasso analysis. Predictor, coefficents per lambda and misclassification error for (a) pure lasso, and (b) elastic net.

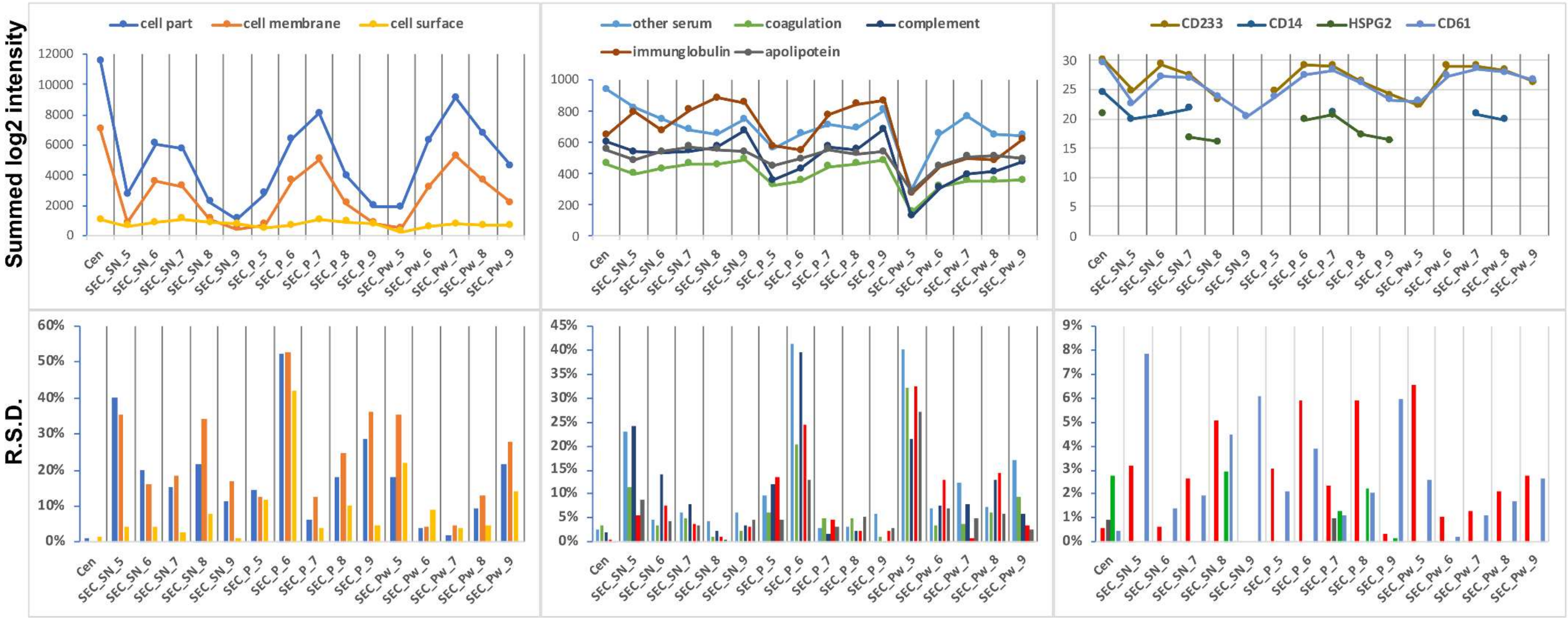

Figure S6. Protein category recoveries by cEV isolation method. The upper row of line graphs illustrates the log2-transformed *Top3* protein intensities summed according to protein category or cell type specific marker protein. Each point represents the mean of three independent replicate analyses performed with each isolation method. The column graphs underneath each line graph show the relative standard deviation to the corresponding data points in the line graphs. The left graphs are for cellular proteins, assumed to represent overall cEV abundance; the middle ones are for proteins of blood plasma representing contamination; and the right graphs are for cell type specific proteins representing erythrocyte (CD233), macrophage (CD14), endothelial cell (HSPG2) and platelet (CD61) origin, respectively. On the x-axes the abbreviations for the cEV isolation protocols are given as explained above in the method section, with the number at the end corresponding to the SEC fraction number.

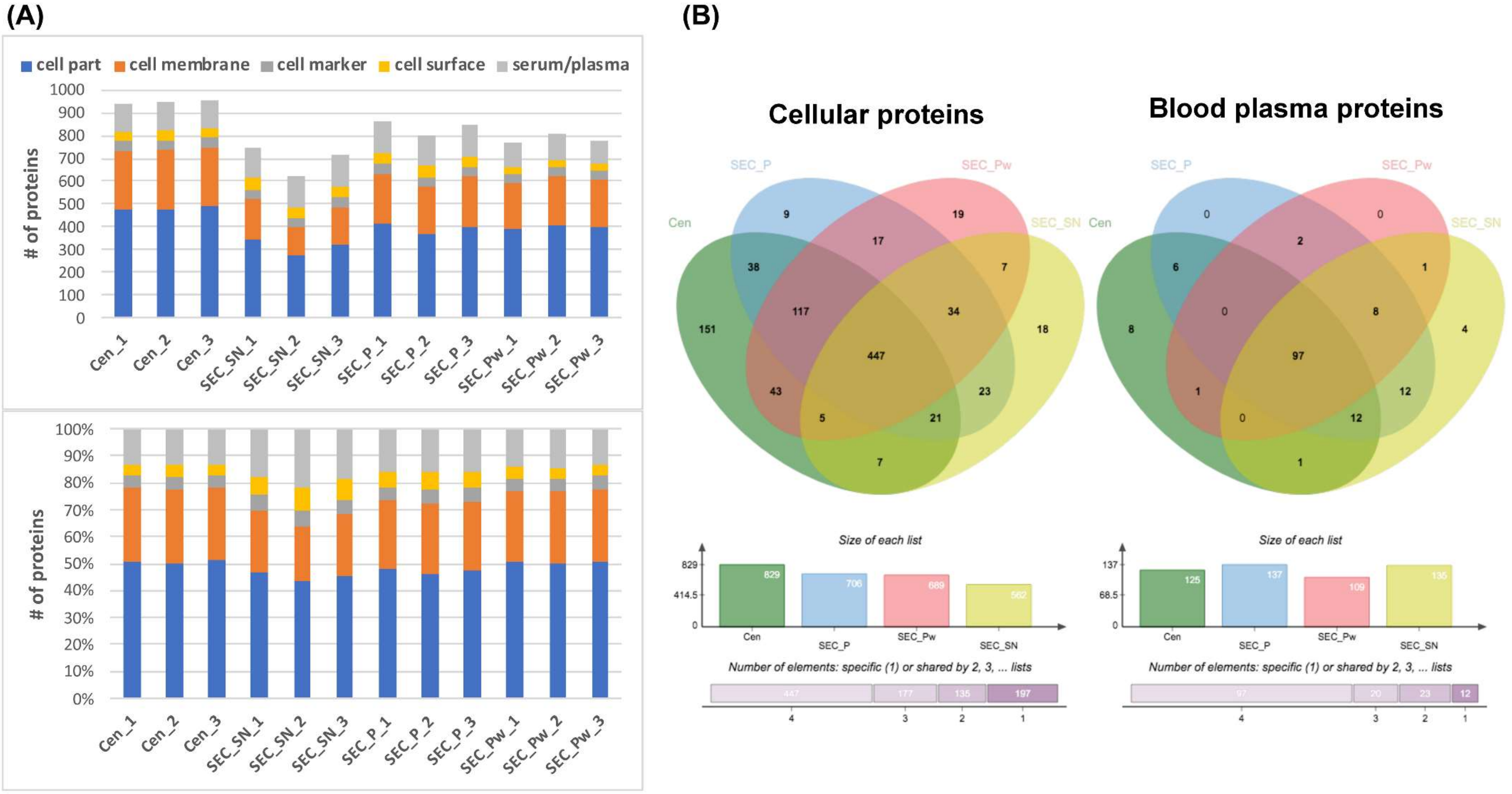

Figure S7. Number of quantified proteins in each cEV isolation replicate. (A) The column graph on top represents the absolute and the one below the relative numbers of proteins quantified to the protein class indicated on top. Each centrifugation replicate is the sum of three repetitive nLC-MS2 analyses for each cEV isolate, while the SEC replicates are the sum of fractions six to eight. (B) The Venn diagrams compare the number of proteins quantified in at least two out of three replicates per method with cellular proteins on the left-hand side and blood plasma contaminants on the right, respectively.

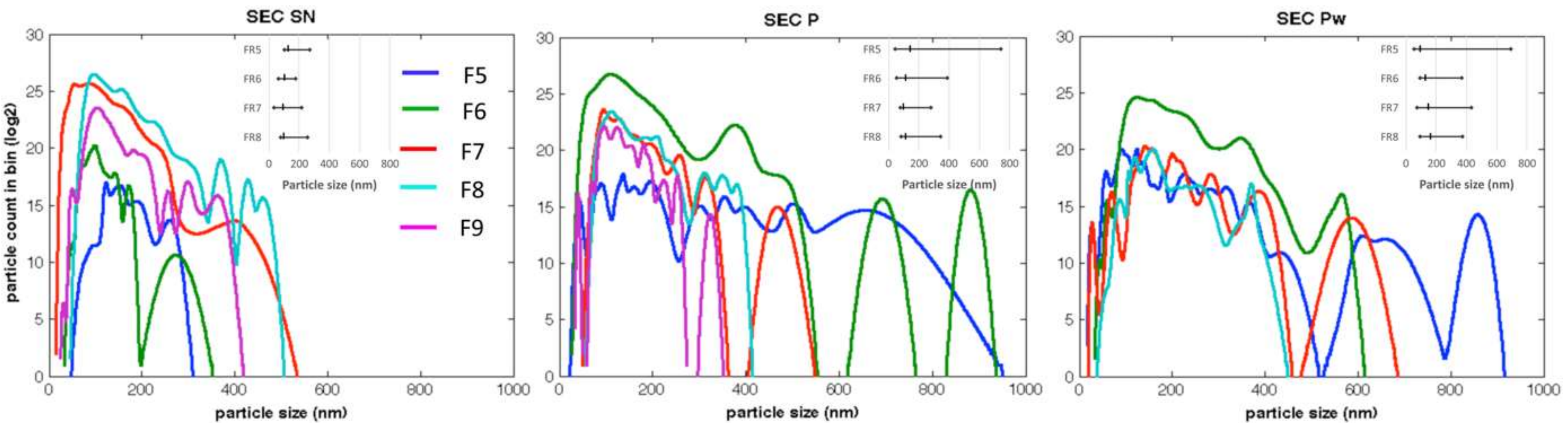

Figure S8. Particle size distribution in SEC fractions. The nano-particle size distribution in fractions five to nine (F5 to F9) were determined from fractions collected by direct SEC separation of PFP (SEC SN, left), after pre-purification of cEV by one high-speed centrifugation (SEC P, middle), or three-times centrifugation/washes (SEC Pw, right). The size distribution for fractions F5 to F8 are given as inserts with the size of the maximum particle count indicated by a vertical line and a horizontal line stretching from left to right from the size where 1% to 99% of all counts were measured.

Supplementary Reference

1. Greganova, E., Heller, M. & Butikofer, P. (2010) A structural domain mediates attachment of ethanolamine phosphoglycerol to eukaryotic elongation factor 1A in Trypanosoma brucei, *PLoS One.* **5**, e9486.
2. Braga-Lagache, S., Buchs, N., Iacovache, M. I., Zuber, B., Jackson, C. B. & Heller, M. (2016) Robust Label-free, Quantitative Profiling of Circulating Plasma Microparticle (MP) Associated Proteins, *Molecular & Cellular Proteomics.* **15**, 3640-3652.
